# Cold Consolidation of Waste Glass by Alkali Activation and Curing by Traditional and Microwave Heating

**DOI:** 10.3390/ma18112628

**Published:** 2025-06-04

**Authors:** Francesco Carollo, Emanuele De Rienzo, Antonio D’Angelo, Paolo Sgarbossa, Luisa Barbieri, Cristina Leonelli, Isabella Lancellotti, Michelina Catauro, Enrico Bernardo

**Affiliations:** 1 Department of Industrial Engineering, University of Padova, 35131 Padova, Italy; paolo.sgarbossa@unipd.it; 2 Department of Engineering ‘Enzo Ferrari’, University of Modena and Reggio Emilia, 41125 Modena, Italy; emanuele.derienzo@unimore.it (E.D.R.); luisa.barbieri@unimore.it (L.B.); cristina.leonelli@unimore.it (C.L.); isabella.lancellotti@unimore.it (I.L.); 3 Department of Engineering, University of Campania ‘Luigi Vanvitelli’, 81031 Aversa, Italy; antonio.dangelo@unicampania.it (A.D.); michelina.catauro@unicampania.it (M.C.)

**Keywords:** alkali-activation, cold consolidation, microwave heating, upcycling, boro-alumino-silicate glass

## Abstract

Despite efforts to recycle, boro-alumino-silicate pharmaceutical glass (BASG) results in a significant portion of glass cullet currently landfilled. Highly contaminated fractions of BASG cullet are largely unemployed because of the presence of metals in their composition that prevents recycling. This waste glass can be eligible to produce sustainable alkali-activated materials (AAMs) reducing at the same time consumption of raw materials and CO_2_ emissions. The ‘weak’ alkaline attack (NaOH < 3 M) determines the gelation of glass suspensions. Condensation reactions occur in hydrated surface layers, leading to strong bonds (Si-O-Si, Al-O-Si, etc.) between individual glass particles. Alkali are mostly expelled from the gel due to the formation of water-soluble hydrated carbonates. Microwave treatment has been implemented on samples after precuring at 40 °C, saving time and energy and achieving better mechanical properties. To improve the stability and reduce the release of glass components into solution, the consolidated monoliths were subjected to boiling/drying cycles. The chemical stability, cytotoxicity and antibacterial behavior of the final products have been investigated with the purpose of obtaining new competitive and sustainable materials. For further stabilization and for finding new applications, the activated and boiled samples can be fired at low temperature (700 °C) to obtain, respectively, a homogeneous foam or a compact material with glass-like density and microstructure.

## 1. Introduction

Glass is theoretically indefinitely recyclable, but there are significant issues on the removal of contaminants from other materials in cullet; contamination can degrade the quality of glass products made from recycled material or even prevent their remelting [1,2]. The recycling process for discarded glasses can be categorized into two main paths: ‘closed-loop’ recycling, consisting of the remelting of waste glass to produce new products that are identical or very similar to the original items and ‘open-loop’ recycling, consisting of the reprocessing of discarded glasses into new products that may differ significantly from their original form [1,2,3].

Circular economy is a crucial foundation for achieving sustainable development goals. The key principle of ‘reduce, reuse, recycle’ addresses global issues such as increasing waste and the depletion of resources [4]. To effectively implement circular practices without causing harmful burden-shifting, reliable data and information are necessary [5]. Over the past two decades, many studies have clarified the environmental impacts of products and their end-of-life scenarios. However, in some instances, recycling appears to be more of a downcycling process than an upcycling one [6].

‘Highly contaminated’ or waste boro-alumino-silicate pharmaceutical glass (BASG) corresponds to the fraction of cullet derived from the crushing of defective vials and syringes directly at the production site, which is contaminated by plastic and metallic heterogeneities. The crushing, in fact, separates coarse fragments of ‘pure’ glass from fine powders, enriched in contaminants, including metallic traces from abrasions of crushing tools. This waste glass cannot be remelted and is therefore currently stored in landfills [3].

In recent years, there has been growing interest in the use of glass waste as raw materials for alkali-activated materials (AAMs) production [7]. The main advantages of AAMs are the drastic reduction in CO_2_ emissions and the possibility to use waste materials as precursors [8,9,10]. During this process, conducted at room temperature, dissolution of aluminosilicate components, promoted by basic nature of the solution, induces the release of ‘inorganic oligomers’, which are molecules made by a few Si^4+^ and Al^3+^ ions bonded together by bridging oxygens provided by terminal-OH groups [3,8]. After this phase, the silicates, borates and aluminates released into solution recombine to develop hydrated alkali alumino-silicate zeolite-like gels [11], which are analogous to those formed by other alkali-activated materials, such as geopolymers [12]. Due to the formation of strong covalent bonds between the released species, the consolidated material is extremely stable and resistant to boiling and acid attack (pH = 5) [12,13].

In cold consolidation, on the other hand, the alkaline attack is weaker (NaOH < 3M) and leads to the formation of strong siloxane bridges involving only the surface of the glass particles [3,14,15]. All glass components in solution form secondary phases, also by interaction with atmosphere (formation of carbonate and hydrate carbonates) [15,16]. The immersion of weak alkali-activated samples in boiling water removes carbonates and secondary soluble phases but leaves the bonds between particle surfaces intact [3,17,18].

Recent studies on metakaolin have shown that microwave heating can be used to dry activated slurries, greatly reducing curing times and energy consumption [19]. It is generally agreed that MW heating is more efficient, prevents the formation of temperature gradients through volumetric heating of the material and, above all, requires less energy [20]. Activated suspensions were left in a 40 °C oven for 24 h and then completely solidified with a five-minute treatment at 450 W in a home microwave. Compared to the ‘cold consolidation’ drying treatment (7 days at 40 °C), microwave curing allows a considerable increase in mechanical strength of the material while maintaining an almost unchanged bulk density.

One limitation of this process is the retention of the obtained material in water. The stagnation in water at 100 °C causes the reaction to reverse with release of glass components into solution and dissolution of the bonds between the surfaces of the BASG particles [21]. This results in partial disintegration of the material with loss of mass and a considerable decrease in resistance after boiling test. To stabilize the activated suspensions, the samples were subjected to successive cycles of boiling and microwave drying. After three boiling/drying cycles, the compression and density resistance values stabilize while the measured pH of the immersion solution is progressively reduced over time.

The present investigation aims to further study and explore the possibilities of using ‘weak’ alkaline activation of glass waste to produce new stable materials with specific characteristics and properties. An industrial waste, in the form of fine BASG powder, was activated with a mild alkaline solution. The resulting slurries were dried through cold consolidation (40–75 °C) combined with microwave heating. Microwave curing allows for a considerable increase in the mechanical strength of the material while maintaining almost unchanged density, saving time and energy [20]. Activated samples withstand successive boiling/drying cycles and are intact even after 30 days of immersion in distilled water.

The release of pollutants and glass components into solution has been investigated by leaching tests conducted on raw and activated materials also at higher molarity. The detection of specific elements (such as heavy metals) is closely related to the stability of the gel phase formed between glass particles during activation process. This phenomenon was considerably reduced by subjecting the material to successive boiling and drying cycles.

Antimicrobial and cytotoxicity studies were conducted on all materials produced to support leaching tests results.

By heat treatment at 700 °C of the samples, it is possible to obtain a homogeneous foam (activated material) or a dense and compact coarse clay (activated and boiled material) [22].

## 2. Materials and Methods

### 2.1. Raw Materials and Preparation

‘BASG waste’, corresponding to BASG glass cullet contaminated by metal particles from needles and grinding tools, was supplied by a company dealing with the manufacturing of pharmaceutical containers and syringes (Stevanato Group, Piombino Dese, Padova, Italy) already in the form of fine powders. These powders were considered, in parallel with particles from dry ball milling of ‘BASG clean’, after sieving below 75 μm. The chemical composition of the starting glasses, provided by the manufacturing company, is reported in Table 1. ‘BASG clean’ derives from the crushing of a batch of vials from a specific supplier, whereas ‘BASG waste’ collected residues of vials and syringes from multiple suppliers, justifying some deviations in chemical formulation from the glass and not from needles and grinding tools.

The finest glass particles were cast into NaOH (reagent grade, Sigma-Aldrich, Gillingham, UK) aqueous solutions, with a molarity of 2.5 M or 5 M, subjected to low-speed mechanical stirring (500 rpm), for 3 h, to obtain a homogeneous slurry. The liquid-to-solid ratio was kept at 0.50. After mixing, the slurries were first poured into silicone molds of dimensions 45 × 40 × 10 mm^3^ and 10 × 10 × 10 mm^3^ (open mold), and then dried at 40 °C in a stove for 24 h. After demolding, the drying was finalized by heating with a domestic microwave oven (Samsung MS23F300EEK, Samsung Electronics Italia S.p.A, Milano, Italy), for 5 min, at 450 W. A summary of the BASG samples produced and characterized in this investigation is given in Table 2.

**Figure 1:**
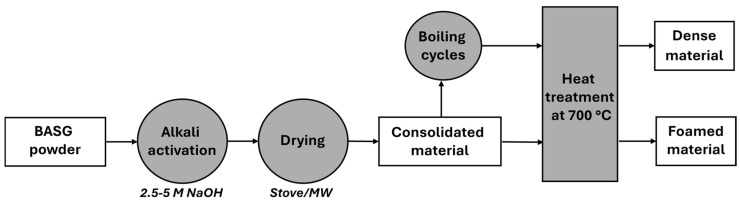
Schematic of the experimental procedure adopted.

To assess the stability of dried samples from waste glass (Waste 2.5 M *MW*), three successive cycles of immersion in boiling water (1 h in distilled water at 100 °C) were performed. After each cycle, the samples were left for 6 h at 75 °C and for 5 min in microwave oven (at 450 W), to ensure complete drying. Selected samples were also considered for firing treatments at 700 °C, for 1 h, in a muffle furnace (BSF-Laboratory Chamber Furnaces by Elite Thermal Systems Limited, Market Harborough, UK) with a heating increase of +5 °C/min. A flowchart of the experimental procedure adopted is shown in Figure 1.

### 2.2. Samples Characterization

The apparent and true densities (ρ_app_ and ρ_true_) were measured by helium pycnometer (Ultrapyc 3000, Anton Paar, Rivoli, Italy), using bulk or finely crushed samples, respectively. Each data point represents the average value of 6 individual tests. The bulk density of the cubic samples (10 × 10 × 10 mm^3^) was evaluated considering the mass measured with an analytical balance and the volume carefully measured with a digital caliper. The density values were then used to compute the amounts of open, closed and total porosity (OP, CP and TP). The mass loss and mechanical resistance of the samples were calculated after each boiling cycle. The compressive strength of the dried objects (before and after boiling) was measured according to the standard UNI EN 826 [23] using a Universal Testing Machine (Quasar 25, Galdabini S.p.a., Cardano al Campo, Italy), operating at a crosshead speed of 0.5 mm/min. Six samples were characterized for each type.

Two cubic samples (10 × 10 × 10 mm^3^) per boiling cycle were immersed in 50 mL of distilled water and the pH of the solution was measured every 24 h for 5 consecutive days by a pH-meter (Edge Series, HANNA Instruments, Villafranca Padovana, Italy) and compared with the value obtained from the activated samples. The mineralogical analysis of powdered samples was carried out using X-ray diffraction (XRD) (Bruker D8 Advance, Karlsruhe, Germany), with CuKα radiation (0.15418 nm), at 40 kV and 40 mA. The scan range was set from 2θ = 10° to 70°, with a step size of 0.05° and a 1 s counting time. Phase identification was performed using the Match!^®^ program package (Crystal Impact GbR, version 1.11f, Bonn, Germany), with reference to data from the Powder Diffraction File (PDF)-2 database (International Centre for Diffraction Data, Newtown Square, PA, USA).

Firing treatment of activated and boiled monoliths was carried out using a ceramic furnace (BSF-Laboratory Chamber Furnaces, Elite Thermal Systems Limited, Market Harborough, UK) at 700 °C for 1 h. Scanning electron microscopy (SEM; Jeol JSM 7600 F, Tokyo, Japan, with 1.2 kV acceleration voltage) investigation was performed to examine the morphology and microstructure of dried samples.

### 2.3. Leaching Tests

Leaching tests were conducted on samples reported in Table 2 at different molarities (NaOH 2.5 M and 5 M). The material that has undergone two successive boiling/drying cycles has also been tested.

Leachate analysis was applied to the activated glass formulations, in order to determine the degree of stabilization of heavy metal components within the matrices by quantifying their dispersion upon prolonged immersion of the samples in water. Leaching tests were carried out following the procedure defined in the EN 12457 European standard [24] for waste characterization: In total, 5 g of material were ground and sieved below 2 mm, placed in Teflon containers, submerged in distilled water (1:10 solid-to-liquid mass ratio), and magnetically stirred for 24 h for maximum exposure of the ground solid to water. Leachates were separated from the solids, acidified to pH = 2 with HNO_3_ aqueous solution and analyzed by means of inductively coupled plasma mass spectrometry (ICP-MS). An ICP-MS iCAPTQ spectrometer model (Thermo Fisher Scientific Inc., Waltham, MA, USA) was utilized to determine ion concentration in diluted leachate samples; dilution was then considered when calculating concentrations in the original leachates.

### 2.4. Microbial and Cytotoxicity Assays

The antimicrobial activity of the samples has been investigated through the slightly modified Kirby–Baur method. To this aim, samples have been finely ground and compressed, thus obtaining 200 mg discs, which have been put in direct contact (after 1 h of UV sterilization) with a solution with 10^5^ CFU/mL of microbial strains (in particular, *Escherichia coli ATCC25922* as Gram-negative bacterium, and *Enterecoccus faecalis ATCC29212* as Gram-positive bacterium) [25]. The bacteria have been plated on their own agar-based solid media and incubated in the presence and absence of the samples. After the microbial growths, the dimension of inhibition halos have been evaluated. Four measurements have been performed for each Petri plate. Data were expressed as mean ± standard deviation (SD).

Human primary keratinocyte cell lines (HaCaT) were cultivated at 37 °C in a humidified environment with 5% CO_2_ in Dulbecco’s Modified Eagle’s Medium (DMEM) supplemented with 10% fetal bovine serum, 50.0 U/mL of penicillin and 100.0 μg/mL of streptomycin. After being seeded at a density of 1.5 × 10^4^ cells/well in 96-well plates, the cells were treated with 1 and 2 mg of samples at 24 and 48 h of exposition. The MTT cell assay was then used to measure the suppression of mitochondrial redox activity (CVI%) [26]. Three replicate measurements were made for each sample amount in separate tests. Data were expressed as mean ± standard deviation (SD).

## 3. Results and Discussion

### 3.1. Mechanical Characterization and Mass Loss

As shown in Table 3, microwave heating allows for a significant increase in the compressive strength of the activated material (18.2 ± 2.4 MPa), compared to that of samples from activation followed by conventional drying (7 days at 40 °C) (10.3 ± 1.8 MPa). The effect is justified by important changes in the bonding of glass particles resulting from the alkaline attack. As shown by Tameni et al. [3], glass particles form a ‘skeleton’, by means of condensation reactions involving surface Si-OH, Al-OH, and B-OH groups (in turn made available by the cleavage of Si-O-Si, Si-O-Al and Si-O-B operated by attacking OH^−^ ions). A SiOH group, at the surface of one particle, may react with a SiOH at the surface of another, leading to the establishment of a Si-O-Si bridge across adjacent particles. Alkali ions cooperate with both atmospheric CO_2_, to form carbonates, and dissolution products (silicates, aluminates and borates), to form a gel. The latter gel is soluble, if highly depolymerized; microwave heating, by promoting carbonate formation [17], reduces the alkali content of the gel and enhances its stability. In other words, the gel is not essential, but it may provide an additional support.

The resistance to immersion in boiling water is a well-known proof of the establishment of a three-dimensional, fully interconnected, molecular structure (‘zeolite-like gel’), for some alkali-activated materials (e.g., for the so-called ‘geopolymers’) [27,28]. The ‘cold consolidation’ process of glass powders, as written above, involves just surface reactions, with most of the mass of glass powders unreacted; despite this, the immersion in boiling water is useful to verify the establishment of strong bonds (e.g., Si-O-Si bridges between adjacent particles), as found in zeolite-like gels [29,30].

Table 3 reports mass, density and compressive strength values after each boiling/drying cycle. The main difference is noted after the first boiling test with the average resistance of the samples decreasing abruptly from nearly 19 MPa to 4.1 ± 1.1 MPa; the compressive strength, however, stabilized with the following cycles. In any case, there was no disintegration of samples; we can say that, observing the remarkable mass loss after the first cycle (8.5 ± 0.7%), followed by much reduced losses (~4%), the compressive strength decreased with the progression of the removal of soluble phases (alkali carbonates and gel). We cannot exclude a contribution to mass loss from mechanical erosion (contacts between samples and container, during boiling operation).

The activation of BASG glass at higher molarity (5 M) led to samples with greatly enhanced density and mechanical strength. The material from waste glass became progressively closer to most alkali-activated materials (AAMs), based on the extensive dissolution of silicate and alumino-silicate feedstock and subsequent gelation of dissolution products [31].

**Figure 2:**
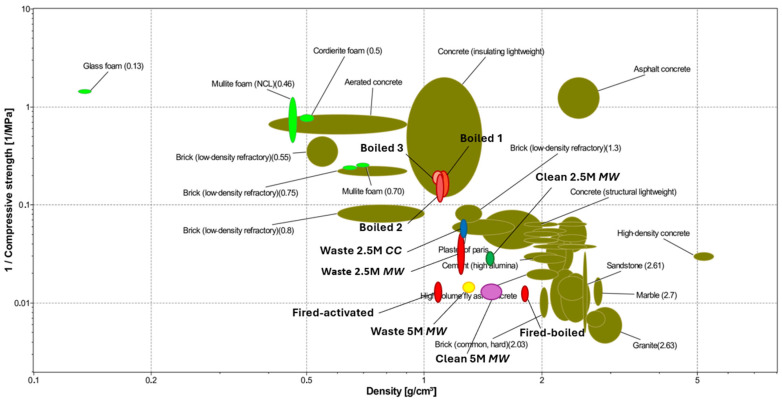
Compressive strength/density trade-off of activated, boiled and fired BASG products (computed by means of CES software package).

A visual representation of the results obtained is provided by Figure 2. The chart displays strength and density of commercial construction materials according to data stored in the latest version of the software Ansys CES (Cambridge Engineering Selector, EduPack, version 2025 R1) with new glass-based cementitious materials introduced as additional entries. Materials with the highest porosity content typically exhibit the lowest strength values (and highest values of 1/compressive strength) and vice versa [32]. Materials from BASG (activated and boiled) are undoubtedly attractive since they exhibit nearly the same density of refractory bricks and lightweight structural concrete, respectively, with improved strength. Microwave heating of BASG appears promising as well, since it provides improved strength combined with almost unchanged density.

### 3.2. Material Stabilization

Alkaline activation of BASG powders does not cause significant changes in the mineralogical structure: the major difference is the appearance of diffraction maxima attributable to sodium carbonate (Na_2_CO_3_, PDF#72-0628) in addition to those already present in the starting material, corresponding to steel particles (in turn coming from abrasion of grinding tools and metallic components, such as needles, joined to glass), leading to weak signals (ferritic matrix, matched by iron, PDF#87-0722, and carbide secondary phase, matched by Fe_3_C, PDF#711174). Carbonates are formed by the interaction of alkali with carbon dioxide in the surrounding environment [10,32].

**Figure 3:**
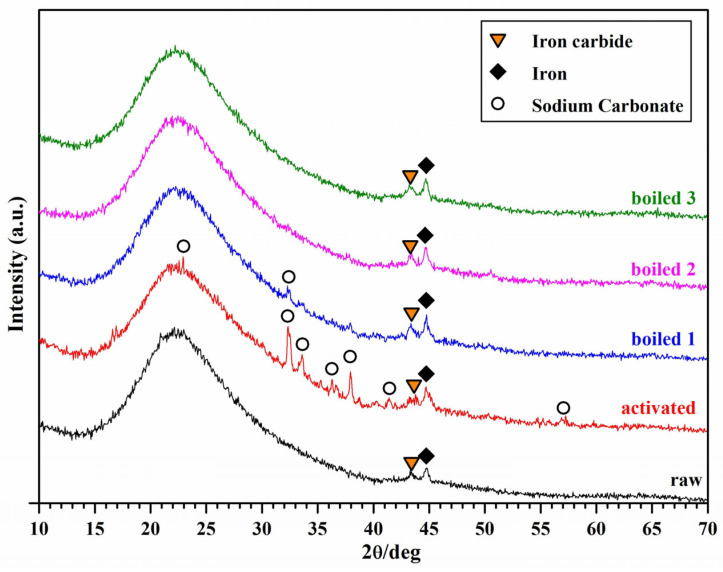
X-ray diffraction analysis of waste boro-alumino-silicate glass in the as-received state, after activation and after each boiling/drying cycle.

Figure 3 shows the XRD patterns comparison of the raw, activated and boiled materials. The samples were completely intact even after 30 days of immersion in water at room temperature.

After the first boiling test (1 h in distilled water at 100 °C) the new crystalline phase is significantly reduced and is completely eliminated after the second boiling. The XRD spectrum of the Boiled 2 sample is perfectly comparable to that of the starting powders. Due to its boro-alumino-silicate chemical formulation, the hardening of glass suspensions has already been attributed to the development of a network structure between particles surfaces that enhances material strength and durability [20,33]. Dried samples did not degrade substantially after immersion in boiling water.

**Figure 4:**
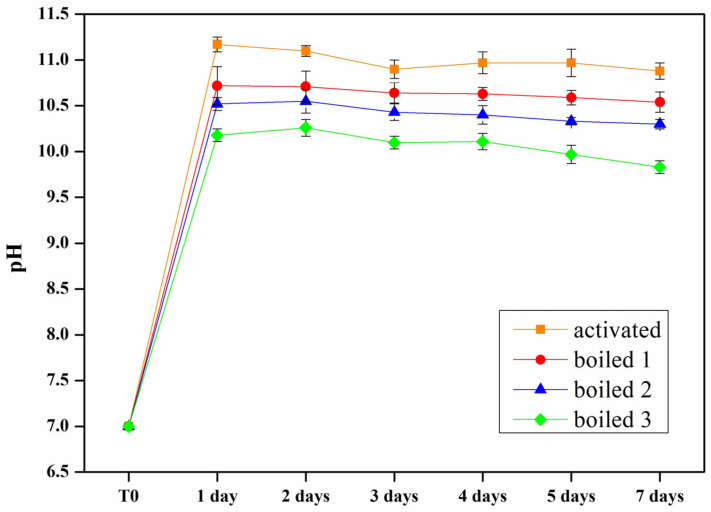
The pH over time of the activated and boiled samples’ immersion solutions. T0 stands for distilled water before sample immersion.

During activation process, alkali are mostly expelled from the gel, according to the formation of water-soluble hydrated carbonates. To evaluate the release of alkaline activator in solution, activated and boiled samples were immersed in 50 mL of distilled water and the pH was measured by pH-meter after 1, 2, 3, 4, 5, and 7 days of immersion. From the values shown in Figure 4, it is noted that the solution of the activated samples is the most alkaline (pH~11); this pH value is due mainly to the dispersion in water of alkali ions that are only partially embedded in the gel surrounding adjacent glass particles [3,21]. The measured values decrease progressively after each boiling/drying cycle (pH < 10 for sample boiled three times). The pH trend over time is approximately constant without evidence of significant increases.

### 3.3. Leaching Tests

**Figure 5:**
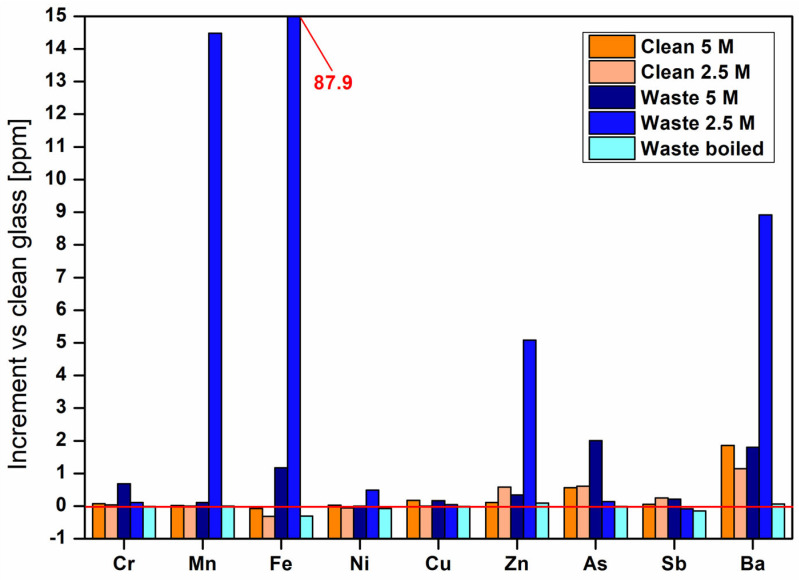
Increase in concentrations of elements released during leaching tests compared to clean glass.

The results of the leaching tests (ppm) differentiated by starting material and alkali activator molarities (2.5 and 5 M), are reported in Table 4. The measured concentrations were then compared with the ones of raw BASG clean (Figure 5). The release of metals in solution is attributable to the unstable gel phase present between the glass particles, which is formed upon alkaline attack and partial dissolution of the precursor and tends to accumulate glass components and expose them to leaching in water [9].

Figure 5 shows the increment in concentrations of the elements released during leaching test by various samples compared to unactivated clean glass (results near 0 mean no variation and negative values a decrease with respect to the starting glass). The measured concentrations are higher for BASG waste samples at lower concentration (2.5 M). The presence of metals pollutants such as Fe, Cr and Mn is due to needle residues from syringes, ground together with discarded glass containers, by the supplier of the starting material. Some metallic traces could derive even from the grinding tools.

Fe and Mn are stabilized by increasing the concentration of the activating alkaline solution. The detection of Ba and amphoteric elements (As and Sb) is due to their use as a stabilizer additive and refining agents in the glass production process [34,35]. The alkaline environment typical of AAM leads to an increase in the leaching of As for waste-glass-containing materials, while Sb seems to be effectively immobilized with the use of 2.5 M solutions. The successive boiling/drying cycles allow for stabilization of the material, considerably reducing the release of these elements into solution (Figure 4).

The enhanced mobilization of As and Sb apparently linked to increased concentration of the activating solution can be attributed to their amphoteric behavior, causing the acquisition of an anionic form for which dissolution is facilitated in alkaline environment, and reversion to cationic form once samples are acidified in preparation for ICP-MS reading.

### 3.4. Antimicrobial Properties and Cytotoxicity Test

**Figure 6:**
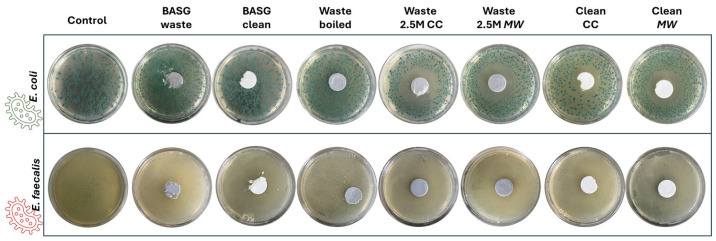
Images of the bacterial growths after incubation with samples.

**Figure 7:**
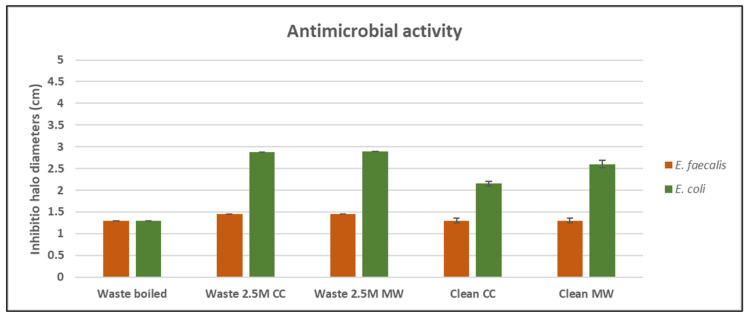
Inhibition halo diameters (cm) values after incubation time of samples in the presence of *E. faecalis* and *E. coli*.

The evaluation of the antimicrobial properties of the samples has been performed on *E. coli* and *E. faecalis*. These bacteria were selected because of their ability to cause nosocomial infections as well as to be indicators of polluted environments [36]. From Figure 6, it is possible to notice that *E. coli* is more sensible to Waste 2.5 M *CC* and *MW*, and Clean *CC* and *MW*, than *E. faecalis.* Indeed, Waste 2.5 M *CC* and *MW*, and Clean *CC* and *MW* showed the highest inhibition halo diameters (see Figure 7). Taking into account the leaching test on possible released heavy metals (see Section 3.4), the increase in inhibition halo diameters of Waste 2.5 M samples can be ascribable to the higher amount of released Fe, Mn and Ba. In fact, according to Nnaji et al. 2024, a high concentration of Fe^3+^ has both inhibitory and toxic effects on *E. coli* [36]. This effect within the alkaline environment coming from the alkali activation of BASG led to higher inhibition halos compared to Clean *CC* and Clean *MW*. In contrast, neither the alkali activation nor *CC* or *MW* treatments led to an increase in antimicrobial properties against *E. faecalis*. Indeed, according to the literature finding, this Gram-positive strain can start growing in alkaline environments (even at pH 10) [37] and in the presence of heavy metals [38].

As regards BASG waste and clean, they showed no effect on both microbial strains. Finally, Waste boiled showed the lowest antimicrobial activity. This is in accordance with our findings since this sample showed both a lowering of pH and heavy metal release.

**Figure 8:**
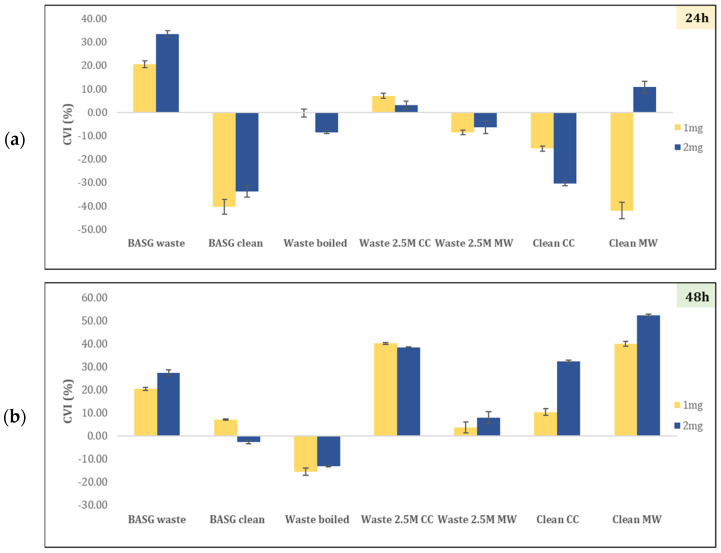
Cell viability inhibition of HaCaT cell line treated with 1 or 2 mg of BASG samples after (**a**) 24 and (**b**) 48 h of incubation time.

A possible cytotoxicity effect on the eucaryotic HaCaT cell line has been investigated at 24 and 48 h of treatment with BASG samples (see Figure 8). Negative values refer to an increase in mitochondrial activity thus indicating a decrease in cell viability inhibition (CVI, %), whilst positive values imply an inhibition of cell viability. Moreover, CVI percentages above 80% are regarded as highly cytotoxic, those between 80% and 60% as strongly cytotoxic, and those between 60% and 40% as moderately cytotoxic. When CVI % is between 40% and 20%, it is slightly cytotoxic, while percentages below 20% are not cytotoxic [39,40]. According to the obtained results, Boiled 2, Waste 2.5 M *CC* and *MW*, and Clean *CC* and *MW* showed no cytotoxic effects within 24 h of exposure. However, after 48 h of exposure, Waste 2.5 M *CC* and Clean *MW* showed moderate cytotoxicity at both lower and higher dosage amounts. It is worth noting that Boiled 2 and Waste 2.5 M *MW* samples showed no cytotoxicity effect. This may suggest that the synthesis parameters of these samples could be the starting point to further enhance the stability of the final products.

### 3.5. Effect of Heat Treatment

Repeated immersion in boiling water of the BASG-based products can be considered an effective solution to the possible release of metals and glass components in solution, allowing one to also recover the alkalis used during the activation phase from the eluate. A further confirmation of the removal of alkali-rich material upon immersion in boiling water was given by the firing treatment at 700 °C.

For the sake of sustainability, the heat treatment was performed far below the temperatures (>850 °C) adopted for the sintering of clay bricks and corresponding to the minimum temperature for viscous flow sintering of boro-alumino-silicate glass [25]. Activated and activated/boiled samples show different mechanical and structural properties after heat treatment at 700 °C (Table 5).

**Figure 9:**
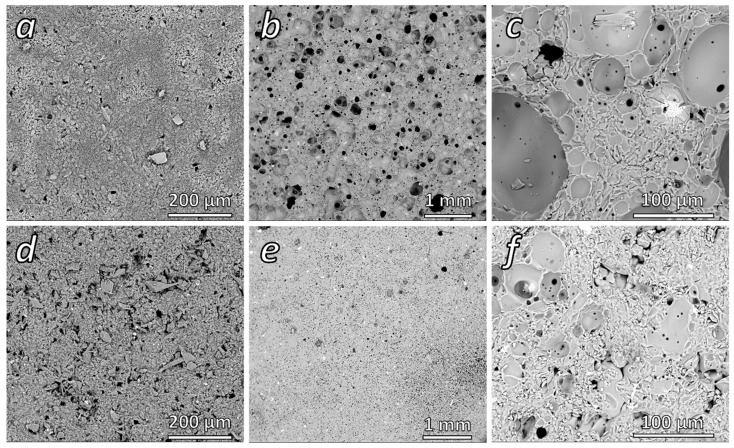
Microstructural images of MW-activated (**a**), activated/fired (**b**,**c**), activated and boiled (**d**) and boiled/fired (**e**,**f**) BASG waste suspensions.

The firing of alkali-activated BASG results in the formation of a lightweight open-celled structure, with porosity exceeding 50% (Figure 9b,c), despite the relatively low temperature. In fact, glass foams are typically produced by gas release, operated by selected additives, in a pyroplastic mass of softened glass well above the temperature required for viscous flow densification [41,42]; the secondary phases in activated BASG acted as ‘softening promoters’ and foaming agents. The alkali-enriched gel reasonably provided a low-viscosity liquid phase [43], supporting the softening; hydrated alkali carbonates, by thermal decomposition, supplied foaming gasses (CO_2_ and water vapour) [44]. Although the bulk density of samples is relatively low (~1 g/cm^3^), the compressive strength (~39 MPa) is comparable to that of ‘dense’ materials, by approaching that of common building bricks and sandstone (Figure 2). The pre-firing materials are also shown in Figure 9a,d.

The removal of secondary phases, with boiling, prevented extensive foaming [44,45]. As reported in Table 5, the resulting material has a higher density (~1.8 g/cm^3^) while the total porosity (TP), mostly open (OP), decreased significantly from ~45 vol% to ~22 vol%. Smaller pores (Figure 9e) were likely due to the water vapor release from condensation reactions of residual Si-OH, Al-OH and B-OH groups. The very limited increase in compressive strength (~40 MPa), compared to the fired activated BASG, was probably due to stress concentration at internal defects, resulting from incomplete sintering, which is visible in Figure 9f (top part) [46]. In any case, firing remains an option for the obtainment of construction blocks, from materials after simple activation or after recovery of alkali and removal of amphoteric elements. Dense products from discarded BASG, both unfired and fired, are suitable for significant practical applications, especially in the construction and environmental sectors (structural concrete and precast elements like tiles, pavers and bricks) [45,46]. On the other hand, porous materials can find applications in thermal and acoustic insulation or as catalytic supports [47].

## 4. Conclusions

This study presents strategies for the exploitation of waste boro-alumino-silicate glass in new sustainable construction materials, according to ‘weak’ alkali activation. Through microwave-assisted cold consolidation of waste glass suspensions, we obtained a stable product with improved mechanical properties, low environmental impact and that is resistant to successive cycles of boiling tests. The chemical and mineralogical stability of activated BASG products was also investigated. The release of metallic cations and glass components in solution, supported by leaching test and due to gel phase instability, has been overcome through successive boiling cycles resulting in a further stabilization of the materials and, eventually, alkali and metal pollutant recovery. In addition, boiled final products show no cytotoxicity and the mechanical strength after successive washing cycles remains comparable with other building materials.

Low-temperature firing (700 °C) of activated and activated/boiled samples confirms the effects of boiling on the elimination of carbonates and other vitreous components from the gel phase. Heat treatments allow for the production of porous or dense and compact bodies, separately, with increased mechanical properties that fall in the strength range of lightweight concrete and common bricks, with lower density.

Overall, the boiling of alkali-activated BASG represents a promising method for recycling waste glass and producing innovative and sustainable materials, while also aligning with circular economy principles by reducing environmental impact and energy consumption. Future research could focus on optimizing the activation conditions, exploring the long-term durability of the developed materials and scaling up the processes for wider application.

## Figures and Tables

**Table 1:** Chemical composition (expressed in wt%) of the starting waste and clean glass.

Oxides	Al_2_O_3_	BaO	CaO	Fe_2_O_3_	K_2_O	MgO	Na_2_O	SiO_2_	TiO_2_	B_2_O_3_	Traces
BASG clean	5.7 ± 0.3	0.08 ± 0.01	1.5 ± 0.3	0.04 ± 0.01	0.14 ± 0.2	-	7.5 ± 0.4	76.1 ± 0.5	-	8.9 ± 0.05	0.04
BASG waste	6.3 ± 0.3	0.54 ± 0.05	1 ± 0.1	3.6 ± 0.5	1.1 ± 0.1	0.11 ± 0.02	6.8 ± 0.5	72 ± 1	0.03 ± 0.01	8.5 ± 0.03	0.02

**Table 2:** BASG-derived samples.

Samples Name	Glass	Alkaline Activator	Consolidation Treatment
Waste 2.5 M *CC*	BASG waste	NaOH 2.5 M (50 wt%)	7 days at 40 °C
Waste 2.5 M *MW*	BASG waste	NaOH 2.5 M (50 wt%)	1 day at 40 °C + 5′ MW (450 W)
Waste 5 M *MW*	BASG waste	NaOH 5 M (50 wt%)	1 day at 40 °C + 5′ MW (450 W)
Clean 2.5 M *MW*	BASG clean	NaOH 2.5 M (50 wt%)	1 day at 40 °C + 5′ MW (450 W)
Clean 5 M *MW*	BASG clean	NaOH 5 M (50 wt%)	1 day at 40 °C + 5′ MW (450 W)

**Table 3:** Mass, density and compressive strength of BASG samples (CC and MW) activated with different molarities and after boiling/drying cycles.

Sample Type	Mass [g]	Bulk Density [g/cm^3^]	Compressive Strength [MPa]
Waste 2.5 M *CC* (*dried*)	0.864 ± 0.023	1.265 ± 0.017	10.3 ± 1.8
Waste 2.5 M *MW* *dried*	0.896 ± 0.015	1.251 ± 0.018	18.2 ± 2.4
*boiled 1st cycle*	0.819 ± 0.020	1.124 ± 0.028	4.2 ± 1.1
*boiled 2nd cycle*	0.789 ± 0.021	1.101 ± 0.014	4.6 ± 1.2
*boiled 3rd cycle*	0.761 ± 0.038	1.089 ± 0.024	3.6 ± 0.4
Waste 5 M *MW* (*dried*)	1.117 ± 0.012	1.309 ± 0.033	35.0 ± 3.1
Clean 2.5 M *MW* (*dried*)	0.981 ± 0.078	1.480 ± 0.024	19.3 ± 2.3
Clean 5 M *MW* (*dried*)	1.032 ± 0.045	1.493 ± 0.083	39.0 ± 5.9

**Table 4:** Results of leaching tests conducted on BASG samples based on clean raw material (clean) and polluted raw material (waste).

Element	Concentrations [ppm]					
	Glass Clean	Clean 5 M	Clean 2.5 M	Waste 5 M	Waste 2.5 M	Waste Boiled
Cr	0.0055 ± 0.0001	0.0760 ± 0.0020	0.021 ± 0.001	0.6900 ± 0.0200	0.116 ± 0.003	0.0007 ± 0.0001
Mn	0.0023 ± 0.0000	0.0178 ± 0.0003	0.001 ± 0.000	0.1160 ± 0.0020	14.500 ± 0.300	0.0058 ± 0.0002
Fe	0.3824 ± 0.009	0.3120 ± 0.0050	0.073 ± 0.009	1.5600 ± 0.0300	88.000 ± 2.000	0.076 ± 0.004
Ni	0.0817 ± 0.002	0.1110 ± 0.0020	0.013 ± 0.001	0.0810 ± 0.0020	0.580 ± 0.010	0.0082 ± 0.0004
Cu	0.0241 ± 0.0004	0.2000 ± 0.0040	0.005 ± 0.001	0.1920 ± 0.0030	0.069 ± 0.001	0.0142 ± 0.0005
Zn	0.0482 ± 0.0006	0.1580 ± 0.0020	0.036 ± 0.002	0.3910 ± 0.0080	5.130 ± 0.090	0.144 ± 0.006
As	0.0618 ± 0.0008	0.6200 ± 0.0100	1.160 ± 0.050	2.0600 ± 0.0400	0.205 ± 0.003	0.057 ± 0.001
Sb	0.1622 ± 0.002	0.2220 ± 0.0030	0.072 ± 0.002	0.3710 ± 0.0060	0.077 ± 0.001	0.0129 ± 0.0008
Ba	0.2393 ± 0.010	2.0900 ± 0.0300	1.250 ± 0.030	2.0400 ± 0.0200	9.200 ± 0.200	0.300 ± 0.020

**Table 5:** Physical and mechanical properties of fired samples.

Sample Type	ρ Geom [g/cm^3^]	ρ App [g/cm^3^]	ρ True [g/cm^3^]	TP [vol %]	OP [vol %]	CP [vol %]	Compressive Strength [MPa]
Fired-activated	1.086 ± 0.015	1.22 ± 0.04	2.29 ± 0.07	52.40 ± 0.65	11.10 ± 1.22	41.38 ± 0.57	38.88 ± 6.23
Fired-boiled	1.809 ± 0.022	2.11 ± 0.04	2.33 ± 0.03	22.29 ± 0.97	14.23 ± 1.07	8.05 ± 1.10	40.16 ± 5.54

## Data Availability

The original contributions presented in this study are included in the article. Further inquiries can be directed to the corresponding authors.
